# On the Chemical and Physiological Balance of Organic Nature

**Published:** 1844-10-01

**Authors:** 


ON THE CHEMICAL AND PHYSIOLOGICAL BALANCE OF ORGANIC NATURE. By *M. J. Dumas* and *M. J. B. Boussingault*, Members of the Institute of France. London, 1844.

THIS Essay, the joint production of Messrs. Dumas and Boussingault, comprises a brief exposition of the grand features in the life of plants and animals considered in a chemical point of view. It presents a variety of new views, calculated to supply general physiology, medicine, and agriculture, with grounds upon which the study of the chemical phenomena that take place in organized beings may be advantageously pursued. Vegetables, animals, man, contain matter in their composition. Whence comes it? What part does it play in their tissues, and in the fluids that bathe them? What becomes of it when death breaks the chain by which its various parts and forms were so closely conjoined? Such are the important and truly interesting questions, which our authors have undertaken to solve in this essay. It cannot fail to excite amazement, when we find that, of all the elements of modern chemistry, organic nature has made use of but three or four; that of those vegetable and animal substances, which are now multiplied almost to infinity, general physiology requires no more than some ten or twelve species; and that all the phenomena of life, so complex in appearance, may be referred in their essence to a single general formula, so simple, that in a few words every thing seems stated, every thing having been recalled to mind, everything foreseen. Numerous results have satisfied the authors that an animal, in a chemical point of view, constitutes a true apparatus of combustion, by which carbonaceous matters, burnt incessantly, are returned to the atmosphere in the shape of carbonic acid; in which hydrogen, burnt incessantly, is returned as water; whence, in fine, free azote is ceaselessly exhaled in the breath, and, in the state of oxide of ammonium, is thrown off in the urine. So that, from the animal kingdom as a whole, carbonic acid, watery vapour, and azote or oxide of ammonium, are continually escaping—simple substances, and few in number, the formation of which is intimately connected with the history of the atmosphere itself. On the other hand, it has been found that vegetables, in their natural and healthy state, decompose carbonic acid incessantly, fixing the carbon, and setting free the oxygen; that they decompose water, seizing on its hydrogen, and disengaging its oxygen as before; lastly, that they either abstract azote directly from the air, or take it indirectly from oxide of ammonium, or nitric acid; thus acting, in every particular, inversely or in opposition to animals. So that, while the animal kingdom constitutes an immense apparatus of combustion, the vegetable kingdom constitutes, in its turn, an immense apparatus of reduction, where carbonic acid decompounded leaves its carbon, water its hydrogen, and oxide of ammonium and nitric acid their ammonium or their azote. If animals incessantly produce carbonic acid, water, azote, and oxide of ammonium, vegetables consequently consume, without cease, oxide of ammonium, azote, water, and carbonic acid. What the one gives to the atmosphere, that the other takes from it; so that it may be said, that plants and animals are the OFFSPRING OF THE AIR; that they are but condensed or consolidated air. Vegetables and animals, therefore, come from the atmosphere, and return to it again; they are true dependents of the air. Vegetables assume from the atmosphere the elements which animals exhale into it; viz. carbon, hydrogen, and azote, or rather carbonic acid, water, and ammonia.

The next question is, how do animals procure the elements which they give to the atmosphere? The simplicity of Nature's laws is truly admirable! Animals always derive their elements primarily from vegetables. Animals do not create any of the truly organic substances; they consume or destroy them; vegetables, on the contrary, habitually create these substances—they destroy but few, and this only for particular and determinate ends. Thus it is in the vegetable kingdom that the great laboratory of organic life is found; it is there that both vegetable and animal substances are compounded; and they are all alike formed at the cost of the atmosphere. From vegetables these substances pass ready-formed into the bodies of herbivorous animals, which destroy one portion of them, and store up another in their tissues. From herbivorous animals they pass ready-formed into the bodies of carnivorous animals, which destroy or lay them up, according to their wants. Finally, during the life of these animals, or after their death, the organic substances in question return to the atmosphere, from whence they originally came, in proportion as they are destroyed. Thus is the mysterious circle of organic life on the surface of the globe completed and maintained! the air contains or engenders the oxidized substances required,—carbonic acid, water, nitric acid, and ammonia. Vegetables, true reducing apparatus, seize upon the radials of these, carbon, hydrogen, azote, ammonium; and with them, they fashion all the variety of organic or organizable matters which they supply to animals. Animals, again, true apparatuses of combustion, reproduce from them carbonic acid, water, oxide of ammonium, and nitric acid, which return to the air to reproduce the same phenomena to the end of time. An agent which acts an undoubted part in all these various phenomena, is the solar light. Without light Nature was without life and without soul: a beneficent God, in shedding light over creation, strewed the surface of the earth with organisation, with sensation, and with thought.

And with respect to the sources whence oxide of ammonium and azotic acid, from which vegetables derive a portion of their food, are derived, these are produced upon the grand scale by the action of those magnificent electric sparks that dart from the storm-cloud, and, furrowing vast fields of air, engender in their course the nitrate of ammonia, which analysis discovers in the thunder-shower. As it is from the mouths of volcanos that the principal food of plants, carbonic acid, is incessantly poured out; so it is from the atmosphere on fire with lightnings, from the bosom of the tempest, that the second and scarcely less indispensable aliment of plants, nitrate of ammonia, is showered down for their behoof.

Scarcely are carbonic acid and nitrate of ammonia formed then LIGHT begins to act on them for new purposes. By its agency carbonic acid yields up its carbon, water its hydrogen, nitrate of ammonia its nitrogen. These elements combine, organic matters are formed, and the earth is clothed with verdure. It is, in fact, from absorbing the light and heat of the sun, that vegetables perform their functions; whilst animals, on their part, engender heat and elicit force in consuming that which vegetables have produced and slowly accumulated. Thus we see the atmosphere presents itself as including the primary materials of all organisation. Volcanos and thunder-storms meet us as the laboratories in which are compounded the carbonic acid and nitrate of ammonia which life requires for its manifestation and extension. Light comes, and with the concurrence of carbonic acid and nitrate of ammonia, the vegetable world, the grand producer of organic matter, is developed. Plants also absorb the solar light which enables them to decompose carbonic acid, water, and ammonia; plants are embodiments of a reducing power of greater virtue than any other that is known, for no other will decompose carbonic acid in the cold.

Next come animals, true consumers of matters, producers of heat and force, true instruments of combustion. In them it is that organised matter acquires its highest expression. In becoming the instrument of sensation and of thought, organised matter is burnt, and in giving out the heat or electricity which constitutes our force, it is destroyed and returned to the atmosphere from which it originally had come. Thus the atmosphere is the mysterious link that connects the two kingdoms (animal and vegetable) together. Vegetables absorb caloric and store up the matter which they have been able to fashion; whilst animals, through which it may be said that organic matter merely passes, burn or consume it, to produce by its means the heat and various forces which their motions turn to profit. The author here likens the vegetable world of the present age, the true storehouse whence animal life is fed, to that other magazine of carbon which we possess in our primæval beds of coal, and which, burnt under the genius of Papin and of Watt, produces carbonic acid, water, heat, motion, we might almost add, life and intelligence. The author here runs through the composition of water, carbonic acid, ammonia, and the air. He concludes this part of the work by stating that the air is a mighty magazine whence plants, for a long time, may draw all the carbonic acid they require for their wants, and where animals, for a still longer period, will find all the oxygen they can consume. So that the atmosphere is a mixture which incessantly receives and incessantly furnishes oxygen, nitrogen, and carbonic acid, by a thousand exchanges, of the nature of which it is now easy to form a right conception.

All plants fix carbon, and all obtain it from carbonic acid, obtained directly from the air by the leaves, or from the soil by the roots, through the decomposition of organic particles and manures in the soil. This carbonic acid, derived by the roots from the soil passing into the trunk, and from thence into the leaves, is eventually exhaled without change into the atmosphere, if no new force intervenes. And such is the case with plants vegetating in the shade, and during the night; the carbonic acid of the soil permeates their tissues and is diffused in the air. It is incorrect to say that plants produce carbonic acid during the night; plants only then transmit unchanged the carbonic acid which their roots have pumped up from the soil. The thing is different, however, when this carbonic acid is in contact with the leaves and green parts, and the light of the sun falls on these; then the carbonic acid disappears; minute bubbles of oxygen are evolved from every point of the leaves, and the carbon is fixed in the tissues of the plant. These green parts of vegetables are also possessed of another property, besides that of decomposing carbonic acid, viz. that of absorbing the chemical rays of the solar light. With respect to the part played by the carbon which has been fixed by plants, it combines with water or its elements, and thus gives origin to substances of the highest consequence in the economy of plants, as for instance, the cellular or the ligneous tissue of plants, or the starch and the dextrine, which are their derivatives. In the same manner do plants decompose water and fix its hydrogen, in order that they may form certain compounds in which this element predominates. The hydrogenous substances thus formed, are employed by plants for various subordinate purposes. Volatile oils serve to defend them from insects; the fat oils serve as materials for combustion, and produce heat at the period of germination; the wax with which the leaves and the fruit are covered, renders them impermeable to water, &c. Thus then, so long as the plant preserves its habitual character, it derives from the sun heat, light, and chemical rays, which it stores up. It receives carbon from the air, takes hydrogen from the water, nitrogen or ammonium from ammonia or nitric acid, and various salts from the soil. With these elementary or mineral substances it fashions organic substances, which accumulate in its tissues. The substances so fashioned and accumulated are either ternary compounds, lignin, starch, gum, sugar, oils or fats; or they are quarternary compounds, fibrine, albumen, caseum, gluten.

So far the vegetable is therefore a constant producer. The animal again constitutes an instrument of combustion, whence carbonic acid is incessantly disengaged, and where, consequently, carbon is incessantly consumed. At the same time that they burn carbon, animals also consume hydrogen. Animals, further, constantly exhale azote; the author insists on the impropriety of admitting an absorption of azote in the course of respiration—we never derive nitrogen from the air; the air, he will have it, is never an aliment for us; all that we do is to take from it the oxygen which is requisite with our carbon to form carbonic acid, with our hydrogen to form water. The nitrogen we exhale, then, proceeds from our food, and from our food only. According to the estimate of M. Lecanu, each individual excretes, on an average, 15 grammes, or nearly 4 drachms, of azote with his urine every day. The azote here is obviously derived from our food, as are the carbon and hydrogen which we burn. The nitrogen escapes in the form of ammonia—this is effected by the urinary secretion, which is a solution of ammonia restored to the earth or atmosphere. And to prevent any injury to the urinary organs from so caustic a substance as ammonia, Nature causes us to excrete urea, this urea is still a carbonate of ammonia; but this carbonate is divested of the proportions of hydrogen and oxygen, which would form water; deprived of it, forms urea, an inert substance. When brought in contact with the air, however, it undergoes fermentation, which restores to it its water, and so converts it into ordinary carbonate of ammonia, an extremely volatile substance, very soluble, and ready to be precipitated in dews and rains; destined therefore to travel from the earth to the atmosphere, from the atmosphere to the earth, until seized upon by the roots of a plant, and elaborated there, it is converted anew into organic matter. Side by side with the urea Nature has placed in the urine infinitely small traces of an albuminous or mucous substance, which undergoes a change when it comes into contact with the air, and becomes a ferment which determines the conversion of the urea into common carbonate of ammonia. If we refer the carbonic acid of the urea to the general phenomenon of animal combustion, to which, indeed, it belongs of right, we shall have ammonia remaining as the characteristic product of the renal secretion. With respect to the lungs and skin then, we have carbonic acid, water and azote; to the urine, ammonia; these are the constant and necessary excretions of the animal body, and these are precisely the elements which vegetables require and turn to use, just as the plant, in its turn, restores to the air the oxygen which the animal had consumed.

We seen then that the carbon and hydrogen, burnt by an animal, and the azote, free or combined, which he exhales, are derived from his food. Viewing the subject of digestion in this way, we shall regard it as much more simple than is usually done. We have seen that an animal creates no organic matter; he is limited to assimilating it, or to expending it by burning. Now digestion is a simple process of absorption. Soluble substances pass into the blood, without alteration for the most part; insoluble substances make their way into the chyle, having been sufficiently comminuted to be imbibed by the lacteals. The object of digestion is to restore to the blood a material fitted to supply our respiration with the $2\frac{1}{2}$ or $3\frac{3}{4}$ drachms of carbon, or an equivalent quantity of hydrogen, which each of us burns in an hour, and also to supply the 15 grains of azote per hour exhaled by the lungs, skin or kidneys.

Amylaceous matters are, therefore, changed into gum and sugar, and these substances are absorbed. Fatty matters are subdivided, formed into an emulsion, and in this way pass into the vessels, to form deposits, which the blood resumes for the purpose of combustion. The neutral azotized substances, fibrine, albumen, and caseum, dissolved at first, and then precipitated, find their way into the chyle, in a state of minute division, or dissolved anew. These animals receive and assimilate, almost unchanged, the neutral azotized substances which they find ready formed in the vegetables or other animals on which they feed; fatty substances they receive from the same sources, and also amylaceous or saccharine substances. These three grand orders of substances, always to be referred for their origin to vegetables, are divided into assimilable products—fibrine, albumen, caseum, fat, which serve for the growth or renovation of organs; and into combustible products, sugar and fats, which are burnt in respiration. Considered in this way the animal machine seems to be the medium between the vegetable world and the atmosphere; it derives all its elements from the former, throws out all its excretions, and finally is itself decomposed into the latter.

Thus then we see that the primitive atmosphere of our globe has formed itself into three great parts—one constituting the atmospheric air of the present time; a second represented by plants; a third by animals. Between these three masses continual exchanges are effected: matter descends from the air into vegetables, penetrates in this way into animals, and returns to the air, according as they apply it to their purposes. Green vegetables constitute the grand laboratory of organic chemistry. They are the agents which, with carbon, hydrogen, azote, water, and oxide of ammonium, slowly form the most complex organic substances. Under the form of heat, or of chemical rays, they receive from the sun the force which enables them to accomplish this great work. Animals absorb the organic substances which plants have formed—they decompound them and bring them back towards the state of carbonic acid, water, azote, and ammonia, in which state they are restored to the air. In burning these organic substances, animals produce caloric, which, radiating into space, goes to supply that which vegetables had absorbed. Thus all that the atmosphere yields to plants, plants yield to animals, animals restore to the air. Eternal round, in which death is quickened and life appears, but in which matter merely changes its place and form! The crude mass of the air, gradually organized in vegetables, passes unchanged into animals, and becomes the instrument of sensation and thought; then, vanquished by this effort, it returns as crude matter to the source from whence it came.

We here conclude our analysis of this interesting little volume. The philosophical views contained in it, we were almost going to say, struck us by their novelty; but we soon recollected that we had seen many of these in Liebig's work. No doubt, the same dish will admit of being sent to table in different forms. We earnestly recommend the perusal of the book to our readers.

